# Thoracic Epidural Analgesia in Major Cancer Surgery: An Update

**DOI:** 10.3390/medicina62091637

**Published:** 2026-08-26

**Authors:** Cyrus Motamed, Marie Josée Caballero

**Affiliations:** 1 Department of Anesthesiology, Gustave Roussy, 94805 Villejuif, France; 2 Faculty of Medicine, Université Paris-Saclay, 94270 Le Kremlin-Bicêtre, France

**Keywords:** thoracic epidural analgesia, cancer surgery, postoperative pain, ERAS

## Abstract

*Background and Objectives*: Postoperative pain after major cancer surgery contributes to pulmonary, cardiovascular, metabolic, and functional complications. Thoracic epidural analgesia [TEA] has traditionally been considered the standard analgesic technique for open thoracic and upper abdominal oncologic surgery, although minimally invasive techniques and ERAS pathways have prompted a re-evaluation of its role. *Methods*: This narrative review summarizes evidence from PubMed and MEDLINE published between 1995 and June 2026, emphasizing randomized trials, systematic reviews, meta-analyses, large observational studies, and selected work by the authors. *Results*: TEA provides superior dynamic analgesia and reduces opioid consumption, facilitating early mobilization, respiratory recovery, and gastrointestinal function. By attenuating sympathetic and neuroendocrine stress responses, it may contribute to reductions in pulmonary complications, ileus, insulin resistance, and delirium, particularly in elderly, frail, sarcopenic, and high-risk patients. Limitations include hypotension, urinary retention, technical failure, and rare but potentially serious complications such as epidural hematoma. In minimally invasive surgery, alternative regional techniques and multimodal opioid-sparing analgesia often achieve comparable postoperative outcomes with fewer hemodynamic effects, supporting selective rather than routine TEA use. Although neuraxial techniques may have immunomodulatory effects and potentially preserve immune function, randomized studies have not demonstrated a reduction in cancer recurrence or an improvement in long-term survival. *Conclusions*: TEA remains an effective analgesic strategy for selected patients undergoing major open thoracic and upper abdominal cancer surgery. In contemporary perioperative practice, its use should be individualized according to surgical invasiveness, patient characteristics and expected postoperative pain. Current evidence supports TEA primarily as a perioperative analgesic and recovery strategy, with no established oncologic benefit.

## 1. Introduction

Postoperative pain remains a major challenge after oncologic surgery despite advances in minimally invasive surgical techniques and multimodal analgesic strategies. Pain is a complex multidimensional phenomenon comprising both sensory-discriminative and affective emotional components, and its intensity may be influenced by psychological factors such as anxiety, depression, catastrophizing, and sleep disturbances [1,2]. Cancer patients frequently experience significant psychological distress associated with their diagnosis, treatment burden, and uncertainty regarding prognosis, all of which may complicate perioperative pain management, increase analgesic requirements, and adversely affect postoperative recovery. Poorly controlled postoperative pain triggers activation of sympathetic and neuroendocrine stress pathways, resulting in increased catecholamine and cortisol release, impaired pulmonary function, delayed mobilization, and metabolic disturbances [3]. These physiological consequences may contribute to pulmonary and cardiovascular complications, insulin resistance, delayed functional recovery, prolonged hospitalization, and impaired quality of life. In addition, severe acute postoperative pain is a recognized risk factor for the development of chronic postsurgical pain, which may persist long after surgical healing and negatively affect long-term survivorship and functional status [4]. Effective postoperative pain control may also facilitate earlier recovery and the timely initiation of adjuvant treatments such as chemotherapy or radiotherapy, which are essential components of multidisciplinary cancer care. Consequently, effective perioperative analgesia remains a cornerstone of contemporary oncologic surgery and Enhanced Recovery After Surgery [ERAS] programs, in which optimal pain control is essential to facilitate early mobilization, respiratory rehabilitation, recovery of gastrointestinal function, and overall postoperative recovery [4,5].

This narrative review was conducted to summarize the current evidence regarding the role and effectiveness of thoracic epidural analgesia in major oncologic surgery. A literature search was performed using PubMed and MEDLINE. Search terms included combinations of “thoracic epidural analgesia”, “cancer surgery”, “oncologic surgery”, “postoperative pain”, “ERAS”, “regional anesthesia”, “pulmonary complications”, and “immune response”. Articles published in English between 1995 and June 2026 were considered. Priority was given to randomized controlled trials, systematic reviews, meta-analyses, clinical practice guidelines, and large observational studies. Because this was a narrative rather than a systematic review, no formal risk-of-bias assessment was performed. However, study selection emphasized methodological quality, clinical relevance, sample size, and current clinical applicability. Additional publications, including selected work by the authors, were included when considered important for understanding the technical aspects of thoracic epidural analgesia, perioperative outcomes, or oncologic implications. Data were synthesized descriptively to provide an updated overview of current clinical practice and emerging evidence in oncology patients, particularly those who are elderly, frail, or sarcopenic.

## 2. Postoperative Pain and Cancer Surgery

Despite advances in minimally invasive surgery and multimodal analgesic strategies, postoperative pain following major oncologic surgery remains a significant clinical problem and is frequently severe after extensive open procedures [5]. Although laparoscopic and robotic approaches generally reduce tissue trauma and analgesic requirements, clinically relevant postoperative pain may still occur because of pneumoperitoneum-related discomfort, diaphragmatic irritation, trocar site trauma, and prolonged operative time. Inadequate pain control is associated with delayed recovery, prolonged hospitalization, impaired mobilization, increased pulmonary and cardiovascular complications, and reduced quality of life [5]. Furthermore, severe acute postoperative pain is a recognized predictor of persistent postsurgical pain and may negatively affect long-term functional outcomes and cancer survivorship [4].

Major cancer surgery remains a cornerstone of curative and/or palliative treatment for solid malignancies. The extent and nature of surgical trauma vary considerably between procedures, with many cancer surgeries still requiring large incisions, extensive tissue dissection, and prolonged operative times despite advances in minimally invasive techniques [6]. Major oncologic procedures involving extensive tissue dissection and manipulation of multiple organs, including esophagectomy, pancreaticoduodenectomy, hepatectomy, radical cystectomy, pelvic gynecologic exenteration, retroperitoneal sarcoma resection, and extensive cytoreductive surgery with hyperthermic intraperitoneal chemotherapy [pseudomyxomas] are associated with particularly severe postoperative pain and frequently require multimodal opioid-sparing analgesia [6,7,8]. In thoracic surgery, rib retraction, intercostal nerve injury, pleural irritation, and chest tube placement generate intense nociceptive input, leading to impaired ventilation and an increased risk of pulmonary complications. Upper abdominal incisions compromise diaphragmatic function, reduce functional residual capacity, and predispose patients to hypoxemia and postoperative respiratory failure. These procedure-specific characteristics contribute to substantial postoperative pain and physiological stress and explain why effective regional analgesia remains an important component of perioperative management, particularly in patients undergoing extensive open surgery [9,10].

Postoperative pain in cancer surgery is predominantly related to extensive tissue dissection, prolonged operative duration, visceral manipulation, and the activation of inflammatory and neuroendocrine stress responses. Surgical trauma initiates a complex cascade of peripheral and central sensitization mechanisms that amplify nociceptive transmission. Tissue injury leads to the release of inflammatory mediators, including prostaglandins, bradykinin, cytokines, hydrogen ions, and neuropeptides such as substance P and calcitonin gene-related peptide. These mediators lower the activation thresholds of peripheral nociceptors, resulting in hyperexcitability and peripheral sensitization. Persistent afferent input to the dorsal horn of the spinal cord activates NMDA receptors and intracellular signaling pathways, leading to central sensitization and long-term neuroplastic changes. This neuroplasticity amplifies pain perception and may persist beyond the immediate postoperative period, increasing the risk of chronic postsurgical pain, highlighting the importance of effective perioperative analgesic strategies in oncologic patients [11].

Cancer patients frequently present with preoperative pain related to tumor infiltration, ischemia, or neuropathic mechanisms. Chronic opioid use is common and may lead to tolerance and opioid-induced hyperalgesia [12,13]. These phenomena complicate postoperative pain management, often requiring escalating opioid doses and increasing the risk of adverse effects such as sedation, ileus, urinary retention, nausea, vomiting, and respiratory depression. Comorbidities such as malnutrition, anemia, sarcopenia, renal or hepatic dysfunction, and immunosuppression further complicate analgesic management. Consequently, postoperative pain following major oncologic surgery is often severe, prolonged, and difficult to treat, making effective analgesic strategies a critical component of perioperative care. The challenge lies not only in achieving adequate analgesia but also in optimizing functional recovery while minimizing opioid-related adverse effects and facilitating adherence to ERAS pathways [14]. Thoracic epidural analgesia [TEA] has historically been considered the reference technique for perioperative and postoperative analgesia following major thoracic and upper abdominal open cancer surgery [14].

By efficiently providing analgesia and achieving sympathetic blockade, TEA reduces systemic opioid requirements, improves respiratory mechanics, enhances gastrointestinal recovery, and facilitates early mobilization [15,16]. These physiological benefits translate into improved functional recovery and reduced postoperative morbidity. TEA interrupts nociceptive transmission at the spinal level by blocking afferent input and producing segmental sympathetic blockade. Continuous infusion of low-concentration local anesthetics, often combined with opioids, enables titration of analgesia while minimizing motor blockade. Patient-controlled epidural analgesia enables individualized dosing and improves patient satisfaction [14]. Modern practice favors dilute local anesthetic solutions to preserve motor function and facilitate early mobilization. Continuous infusion and programmed intermittent bolus techniques improve longitudinal spread of local anesthetics within the epidural space and enhance block quality while reducing total drug consumption [17,18]. However, the emergence of minimally invasive surgical techniques, multimodal opioid-sparing analgesia, and newer regional anesthetic approaches has prompted a re-evaluation of the routine use of TEA in all cancer patients. Consequently, current practice increasingly emphasizes individualized selection of analgesic techniques according to the type of surgery, patient comorbidities, expected postoperative pain burden, and recovery objectives [16,17,18,19].

## 3. Local Anesthetics, Neuraxial Analgesia and Cancer

### 3.1. Experimental and Preclinical Evidence

Lidocaine is thought to exert a direct inhibitory effect on tumor growth; a similar findings have been reported with ropivacaine. Intravenous lidocaine may also potentiate the effects of chemotherapy. Using a breast cancer model, Li et al. [20] found that lidocaine enhanced the antitumor action of cisplatin, resulting in an increased antiproliferative effect. A dose-dependent DNA demethylation effect has also been described, which may prevent the silencing of tumor suppressor genes. These observations are supported by growing experimental evidence suggesting that lidocaine can modulate tumor-related signaling pathways, immune interactions, and metastatic processes [21].

However, these findings cannot be generalized to all local anesthetics, as available data remain heterogeneous and are largely derived from preclinical studies. Different agents appear to exhibit distinct biological profiles: lidocaine is the most extensively investigated and has demonstrated epigenetic and immunomodulatory effects, whereas moderate evidence supports ropivacaine’s anti-migration and pro-apoptotic activities, and bupivacaine has demonstrated marked cytotoxicity in vitro [22]. Importantly, many reported anticancer effects occur at concentrations higher than those achieved during routine clinical use or require prolonged exposure that are not representative of standard perioperative practice [22]. Consequently, the clinical relevance of these findings remains uncertain despite a compelling mechanistic rationale.

### 3.2. Observational Clinical Evidence

From a clinical perspective, robust evidence demonstrating improved oncologic outcomes with any local anesthetic is still lacking. Although some meta-analyses suggest that regional anesthesia techniques may be associated with improved survival or reduced recurrence in certain cancers, the results are inconsistent and largely based on observational data [21]. In this context, neuraxial strategies such as thoracic epidural anesthesia [TEA], by attenuating the surgical stress response, reducing opioid requirements, and preserving immune function, have been hypothesized to influence cancer recurrence; however, current evidence remains inconclusive [22].

Some studies have proposed that regional anesthetic techniques such as TEA may reduce the risk of cancer recurrence by modulating the perioperative environment in ways that are biologically unfavorable to tumor dissemination [23,24]. Major surgery triggers a cascade of neuroendocrine and inflammatory responses characterized by increased sympathetic activity, the release of catecholamines and cortisol, and the production of pro-inflammatory cytokines. TEA attenuates this stress response and may help preserve immune surveillance [24]. Clinical studies have demonstrated less suppression of NK cell activity and lower levels of protumorigenic cytokines when epidural techniques are combined with general anesthesia [25]. Additionally, TEA may reduce opioid exposure, thereby limiting opioid-associated immunomodulation, as described in experimental models [25].

Beyond immunologic preservation, TEA may contribute to a more favorable perioperative environment through improved tissue perfusion, reduced hypoxia, and facilitation of recovery pathways that may allow for earlier initiation of adjuvant treatments when required [26]. However, interpretation of observational findings remains difficult due to substantial heterogeneity in cancer type, surgical procedure, anesthetic management, and study methodology, as well as the potential for residual confounding and selection bias.

### 3.3. Randomized Clinical Evidence

Despite compelling biological hypotheses and encouraging observational findings, randomized evidence remains largely negative [27,28,29,30]. Xu et al. demonstrated that epidural anesthesia–analgesia did not significantly improve recurrence-free survival following lung cancer surgery [29]. Falk et al. similarly found no improvement in disease-free survival after colorectal cancer surgery when epidural analgesia was compared with intravenous analgesia [31]. Furthermore, large meta-analyses incorporating both randomized and observational studies have failed to demonstrate a consistent reduction in cancer recurrence or improvement in overall survival attributable to regional anesthesia alone [23,29,30].

Taken together, current evidence does not support the routine use of TEA as an oncologic intervention. Rather, TEA should be regarded primarily as a perioperative analgesic technique that optimizes recovery and physiological function, while any potential influence on long-term cancer outcomes remains an area of ongoing investigation [27,30,31,32], Table 1.

## 4. Epidural Analgesia Principles and Practical Management

### 4.1. Epidural Catheter Placement and Management

Thoracic epidural analgesia is generally performed preoperatively under strict aseptic conditions and remains the reference neuraxial technique for major thoracic and upper abdominal surgery. Although the technical principles of epidural placement are well established, successful catheter placement requires considerable expertise because of the anatomical complexity of the thoracic spine [39]. Epidural catheters are typically inserted between T4 and T8 for thoracic procedures and between T7 and T10 for upper abdominal surgery, depending on the anticipated dermatomal distribution of postoperative pain. Following placement, catheter function and block efficacy should be assessed regularly using sensory mapping, pain scores and motor blockade assessment, while anticoagulation management should follow established neuraxial safety guidelines.

Epidural failure may result from several mechanisms, including incorrect catheter positioning outside the epidural space, inadequate segmental spread of local anesthetic, intravascular or intrathecal migration, kinking or coiling of the catheter, or patient-specific anatomical variations such as scoliosis, spinal stenosis, or prior spinal surgery [40]. Reported failure rates remain clinically relevant and may negatively affect postoperative recovery if not recognized and managed promptly [40,41].

Ultrasound guidance has been increasingly explored as an adjunct to traditional landmark-based techniques to improve the accuracy of epidural placement. This modality is especially useful for identifying anatomical landmarks and estimating the depth to the epidural space in patients with obesity, advanced age, spinal deformities, or poorly palpable landmarks. Pre-procedural ultrasound scanning has been shown to improve midline identification, optimize needle insertion angle, reduce the number of needle passes, and decrease the risk of accidental dural puncture [42]. Additionally, ultrasound may enhance operator confidence and procedural efficiency, particularly for trainees or in technically difficult cases. However, despite these potential advantages, evidence remains mixed regarding the superiority of ultrasound-guided techniques over conventional landmark-based approaches when performed by experienced practitioners [43]. Current evidence suggests that ultrasound-assisted epidural placement is most beneficial in patients with obesity, advanced age, scoliosis, previous spinal surgery, poorly palpable landmarks, or anticipated difficult neuraxial access. It may also improve procedural success during training and reduce the number of needle passes required. Although superiority is less evident among highly experienced practitioners, ultrasound represents a valuable adjunct for selected patient populations and difficult procedures [42,43].

Thoracic epidural analgesia is most commonly initiated preoperatively or at induction and continued postoperatively, rather than used only postoperatively, because intraoperative TEA [combined with general anesthesia] provides more effective blockade of nociceptive input, blunts the surgical stress response, and improves postoperative pain and pulmonary outcomes compared with postoperative analgesia alone. Pre-incisional blockade allows segmental analgesia to begin before tissue trauma and may reduce early postoperative pain scores and opioid consumption compared with postoperative-only initiation [16,44,45]. Meta-analyses of studies of major thoracic surgery demonstrate that perioperative TEA [started preoperatively or intraoperatively and continued postoperatively] reduces postoperative pulmonary complications and pain compared with systemic opioids. Data from randomized clinical trials also show that TEA initiated prior to surgical incision provides better early analgesia and lower pain scores than delayed or postoperative-only TEA, supporting the concept of preemptive analgesia [44,45]. While postoperative TEA alone still provides effective analgesia compared with systemic opioids, combining intraoperative use with postoperative epidural infusion is considered optimal for enhanced recovery and attenuation of the stress response [16,46]. Nevertheless, for minimally invasive thoracic procedures such as video-assisted thoracoscopic surgery [VATS], alternative regional techniques including thoracic paravertebral block and erector spinae plane block may provide comparable analgesia with fewer hemodynamic adverse effects and are increasingly incorporated into ERAS pathways [47].

### 4.2. Management of Epidural Failure and Rescue Analgesia

In the post-anesthesia care unit [PACU], failure of TEA should be managed using a structured rescue approach aimed at achieving rapid pain control while avoiding hemodynamic instability and excessive opioid exposure. Initial management includes immediate assessment of catheter function [dermatomal sensory level, bilateral spread, motor block, pump settings, and catheter integrity] and exclusion of intrathecal or intravascular migration [48]. If partial or unilateral block is present, rescue measures include epidural bolus dosing with dilute local anesthetic ± opioid, patient repositioning, or careful catheter withdrawal by 1–2 cm to correct malposition [48]. In the event of complete failure or contraindications to further epidural administration, systemic multimodal analgesia should be promptly instituted, combining intravenous acetaminophen, NSAIDs [if not contraindicated], and cautious opioid titration, preferably via patient-controlled analgesia [18]. Severe acute pain in the PACU following epidural failure can also be treated with a combination of remifentanil and incremental morphine titration in order to shorten the time of acute stress and pain [49].

Regional rescue techniques such as thoracic paravertebral block or erector spinae plane [ESP] block can be safely performed in the PACU and provide effective unilateral or bilateral analgesia with a lower risk of hypotension compared with TEA, particularly after thoracic and upper abdominal cancer surgery [37,50]. In abdominal surgery, transversus abdominis plane or quadratus lumborum blocks may serve as adjuncts, although visceral analgesia may be less reliable [33]. If pain remains uncontrolled or epidural-related complications are suspected [e.g., hypotension, neurologic deficit], the epidural catheter should be removed and alternative analgesic strategies continued, with early involvement of the acute pain service [51]. This structured approach allows for rapid correction of epidural failure while maintaining effective pain control and minimizing the disruption of recovery pathways.

### 4.3. Removal of Epidural Catheter

Safe removal of a thoracic epidural analgesia catheter requires careful consideration of patient-specific factors, particularly concurrent anticoagulant or antiplatelet therapy, to minimize the risk of complications such as epidural hematoma, catheter breakage, or catheter entrapment. Standard practice guidelines recommend gentle, continuous traction without excessive force, and documented strategies include adjusting the patient’s position [e.g., lateral decubitus or the same position used during insertion] and reassessing resistance before further attempts to reduce the risk of damage or entrapment. In patients receiving anticoagulants such as low-molecular-weight heparin [LMWH], consensus guidelines recommend withdrawing the epidural catheter only after an appropriate interval from the last anticoagulant dose [e.g., ≥12 h after prophylactic LMWH administration] and before subsequent anticoagulant administration [52]. Particular vigilance is required during the hours immediately following catheter removal, as early recognition of neurological symptoms remains critical for the diagnosis and management of epidural hematoma.

In an ICU setting, removal should only be considered when the patient is sufficiently awake and not deeply sedated, enabling providers to perform an adequate neurological assessment after removal if necessary. If unusual resistance is encountered, imaging [e.g., CT scan or MRI] may help assess the catheter’s position and guide further management or facilitate non-invasive extraction, while retained fragments are managed conservatively if asymptomatic or surgically in symptomatic cases to prevent late complications [53]. Adherence to established neuraxial safety recommendations and systematic post-removal surveillance remains essential for ensuring patient safety and minimizing rare but potentially devastating complications.

Another recognized complication of thoracic epidural analgesia [TEA] is post-dural puncture headache [PDPH], which occurs following accidental puncture of the dura mater. Leakage of cerebrospinal fluid [CSF] through the dural defect results in reduced CSF volume and intracranial hypotension, causing traction on pain-sensitive intracranial structures. The incidence of inadvertent dural puncture during neuraxial techniques is approximately 1%, and PDPH develops in approximately 60–80% of affected patients, making it an important source of morbidity despite its overall low frequency. Clinically, PDPH is typically positional and may be accompanied by neck stiffness, nausea, photophobia, or auditory symptoms [54]. Diagnosis is primarily clinical and should be considered in any patient presenting with characteristic postural headache following spinal tap. Initial management is primarily conservative and/or symptomatic and includes adequate hydration, oral analgesics, and 24-h caffeine administration [oral or intravenous] which may provide symptomatic relief through cerebral vasoconstriction [55]. When symptoms are severe, cause significant functional limitation, or fail to respond to conservative therapy, an epidural blood patch remains the treatment of choice and the current standard intervention, providing rapid symptom relief by sealing the dural leak and restoring CSF dynamics [56]. Early recognition and appropriate management are important because persistent symptoms may delay mobilization, prolong hospitalization, and negatively affect postoperative recovery.

## 5. Benefits and Limitations of TEA

### 5.1. Analgesic Efficacy and Impact on Postoperative Recovery

Multiple randomized trials and meta-analyses demonstrate that TEA provides superior analgesia compared with systemic opioids following thoracic and upper abdominal surgery [16]. This advantage is particularly evident during movement, coughing, and physiotherapy, when effective pain control is essential for postoperative recovery. Effective dynamic analgesia facilitates participation in respiratory exercises and early mobilization, which are key components of enhanced recovery pathways [19]. TEA significantly reduces systemic opioid requirements, decreasing nausea, vomiting, sedation, pruritus, urinary retention, and postoperative ileus. Reduced opioid exposure may also decrease the risk of persistent opioid dependence after surgery [19]. These benefits are most consistently observed after major open thoracic and upper abdominal procedures, whereas the magnitude of benefit may be less pronounced in minimally invasive surgery, where overall pain intensity is generally lower [19].

Respiratory benefits are central to the rationale for TEA. By providing effective analgesia without causing significant respiratory depression, TEA facilitates deeper inspiration, more effective coughing, and improved participation in physiotherapy, translating into better oxygenation and fewer pulmonary complications [16]. These benefits are particularly relevant in patients with limited pulmonary reserve, including those with chronic obstructive pulmonary disease [COPD], restrictive lung disease, a history of smoking, or prior thoracic radiotherapy. By minimizing systemic opioid administration, TEA further reduces the risk of opioid-induced hypoventilation. However, although improved respiratory outcomes have been demonstrated in many studies, the magnitude of benefit varies according to surgical approach, patient comorbidities, and concomitant ERAS interventions. Consequently, careful patient selection remains essential to maximize the clinical value of TEA in contemporary perioperative practice [16,57].

Selected patients with severe COPD may benefit from TEA even during minimally invasive major abdominal surgery. Although laparoscopic approaches generally reduce postoperative pain and opioid requirements, patients with limited respiratory reserve may still experience clinically significant hypoventilation when exposed to systemic opioids. Observational studies have reported reduced pulmonary complications and improved perioperative outcomes in high-risk respiratory patients receiving neuraxial analgesia [58,59,60]. However, the quality of evidence remains limited, and the magnitude of benefit may vary according to the surgical procedure, patient characteristics, and perioperative care pathway. In routine practice, TEA may be selectively indicated for COPD patients undergoing minimally invasive surgery when opioid minimization is a priority, particularly in individuals with severe respiratory impairment or elevated pulmonary risk [61]. These considerations support an individualized approach rather than routine application of TEA in all minimally invasive procedures.

Sympathetic blockade associated with TEA improves splanchnic blood flow and gastrointestinal motility. Combined with reduced opioid exposure, this facilitates earlier return of bowel function and tolerance of oral intake [60]. Postoperative ileus remains a major contributor to prolonged hospitalization following major abdominal cancer surgery, and TEA has been associated with shorter times to first flatus and bowel movement in several studies [62]. Early enteral feeding improves nutritional status, preserves gut integrity, and reduces infectious complications [63]. These gastrointestinal benefits are particularly relevant after major open abdominal surgery, where postoperative ileus remains common. However, in minimally invasive surgery, the incremental benefit of TEA over multimodal opioid-sparing analgesia may be less pronounced because laparoscopic and robotic techniques themselves contribute to accelerated gastrointestinal recovery. Consequently, the choice between TEA and alternative regional analgesic techniques should be individualized according to surgical invasiveness, anticipated pain severity, and ERAS objectives [60].

Early mobilization is a cornerstone of Enhanced Recovery After Surgery [ERAS] pathways [63]. Effective analgesia is a major component of successful ERAS implementation, as pain represents a significant barrier to ambulation, respiratory physiotherapy, and early resumption of oral intake [64,65]. Thoracic epidural analgesia has historically been a central component of ERAS protocols for open abdominal and thoracic surgery. By providing superior dynamic analgesia, TEA enables patients to sit, stand, and ambulate safely during the first postoperative days, thereby reducing venous thromboembolism risk, muscle deconditioning, insulin resistance, and adverse pulmonary events. These benefits are particularly relevant in oncology patients, who frequently present with frailty, sarcopenia, and limited physiological reserve [64,66,67]. In patients undergoing major open thoracic or upper abdominal surgery, TEA continues to provide important advantages by combining effective pain control with improved respiratory function and facilitation of postoperative recovery.

### 5.2. Other Potential Systemic and Physiological Effects

The following effects are biologically plausible, but their clinical relevance remains variable, or insufficiently demonstrated.

Renal perfusion may be favorably influenced by TEA through attenuation of stress-related vasoconstriction and reductions in catecholamine release. However, direct renal protective effects remain insufficiently demonstrated, and current evidence should be interpreted cautiously. This potential effect may be particularly relevant in oncologic patients with borderline renal function, advanced age, or prior exposure to nephrotoxic chemotherapy. In addition, reduced opioid consumption may indirectly contribute to improved postoperative recovery and a lower incidence of opioid-related adverse effects, although a specific protective effect against acute kidney injury has not been consistently demonstrated [68].

Postoperative delirium represents a major complication in elderly oncology patients, as it is associated with increased morbidity, prolonged hospitalization, and long-term cognitive decline [69]. Risk factors include poorly controlled pain, high-dose opioids, sleep deprivation, infection, and metabolic disturbances. By providing effective analgesia and reducing systemic opioid exposure, TEA may contribute to lower rates of postoperative delirium in selected patients. However, available evidence remains limited, and randomized studies have not consistently demonstrated a definitive protective effect [70]. Consequently, delirium prevention should not be considered a primary indication for TEA, although improved pain control and opioid sparing may represent potentially beneficial secondary effects.

Thermoregulation is another physiologic domain influenced by TEA. Sympathetic blockade promotes peripheral vasodilation and the redistribution of core heat, increasing the risk of intraoperative hypothermia. However, TEA also reduces postoperative shivering and metabolic oxygen consumption, which may be beneficial in patients with limited physiological reserve. Although these thermoregulatory effects are usually clinically manageable, maintenance of perioperative normothermia remains important within modern ERAS pathways to optimize recovery and reduce complications [71,72].

From a metabolic perspective, attenuation of the surgical stress response induced by TEA limits postoperative insulin resistance and hyperglycemia. Hyperglycemia is a recognized risk factor for surgical site infection and impaired wound healing. By reducing cortisol and catecholamine secretion, TEA may contribute to improved glycemic control, particularly in patients with diabetes or metabolic syndrome. These metabolic effects may support postoperative recovery; however, the magnitude of their clinical impact remains incompletely defined.

### 5.3. Patient Selection and Surgical Context

With the increasing adoption of minimally invasive surgical techniques, the routine use of TEA within ERAS programs has been questioned [73]. Laparoscopic and robotic approaches are associated with reduced parietal trauma and lower postoperative pain intensity compared with open surgery. In this context, several randomized trials and meta-analyses demonstrate that while TEA may improve early postoperative pain scores during the first postoperative hours, it does not consistently shorten hospital stay or accelerate recovery of bowel function compared with multimodal opioid-sparing analgesia [36]. Furthermore, epidural-related adverse effects such as hypotension, urinary retention, pruritus, and lower limb weakness may delay mobilization and potentially counteract ERAS objectives. Consequently, the balance between benefit and risk differs substantially between open and minimally invasive surgery. Whereas TEA remains particularly valuable for extensive open thoracic and upper abdominal procedures, alternative regional techniques such as thoracic paravertebral block, erector spinae plane block, and multimodal analgesic strategies frequently provide comparable recovery outcomes after minimally invasive surgery with fewer hemodynamic adverse effects [36,47].

Current ERAS recommendations therefore favor a selective rather than routine approach to TEA, reserving its use for patients most likely to benefit, including individuals at high pulmonary risk, those with severe preoperative pain, opioid tolerance, frailty, or procedures associated with substantial postoperative pain burden. Analgesic selection should ultimately be individualized according to surgical invasiveness, patient comorbidities, expected recovery trajectory, and institutional expertise [68].

### 5.4. Limitations and Risks of TEA

Hemodynamic instability represents one of the principal limitations of TEA. Segmental sympathetic blockade produces vasodilation, reduced systemic vascular resistance, and decreased venous return [34]. Hypotension is particularly common in elderly patients, those with pre-existing autonomic dysfunction, hypovolemia, or concomitant antihypertensive therapy [34]. Because significant hypotension may compromise organ perfusion, particularly in patients with limited cardiovascular reserve, careful patient selection and perioperative monitoring are essential. Careful preoperative assessment, goal-directed fluid therapy, and judicious vasopressor support are essential to maintaining hemodynamic stability while preserving analgesic benefits [34]. The use of low-concentration local anesthetic solutions and incremental dosing strategies can minimize excessive sympathetic blockade and reduce the incidence of hypotension [74]. The clinical relevance of epidural-related hypotension varies according to patient characteristics, surgical complexity, and perioperative management, emphasizing the need for an individualized risk–benefit assessment when considering TEA.

TEA is associated with several other limitations that must be weighed against its analgesic benefits, such as urinary retention, pruritus, nausea, and lower limb weakness, which can delay mobilization and counteract enhanced recovery [ERAS] objectives. With the increasing adoption of minimally invasive surgical techniques, the routine use of TEA within ERAS programs has been questioned [34]. In some comparative settings, multimodal analgesia has been associated with fewer hypotensive episodes and vasopressor requirements compared with TEA, raising concerns about its risk–benefit profile in these populations [33,75,76,77]. Technical challenges also limit the widespread application of TEA, as placement can be difficult in patients with obesity, spinal deformities, or prior spine surgery, and catheter failure or incomplete blockade remain clinically relevant issues despite experienced operators. Furthermore, alternative regional techniques such as thoracic paravertebral block and erector spinae plane block may provide effective analgesia with fewer hemodynamic adverse effects in selected patients, particularly after minimally invasive surgery [35].

The use of neuraxial analgesia is further constrained by coagulation abnormalities and the increasing perioperative use of anticoagulant and antiplatelet medications, which increase the risk of rare but serious complications such as epidural hematoma. Although large retrospective series suggest that major complications are uncommon in experienced centers, the potential for serious adverse events necessitates careful patient selection, adherence to safety guidelines, and close monitoring.

The growing availability of alternative regional techniques and multimodal analgesic strategies has substantially influenced contemporary perioperative practice. Thoracic paravertebral block, erector spinae plane block, quadratus lumborum block, TAP block, and multimodal opioid-sparing analgesia have demonstrated efficacy in selected surgical settings and may offer advantages such as greater technical simplicity or reduced hemodynamic effects [31,33,35,48]. These alternatives are best considered within the broader individualized approach to analgesic selection described above, particularly in minimally invasive surgery where they may provide comparable recovery outcomes. As a result, current practice increasingly favors individualized analgesic strategies rather than routine TEA use in all major oncologic procedures, particularly in minimally invasive surgery where alternative approaches may provide comparable recovery outcomes.

## 6. Indications of TEA in Different Major Surgical Cancer Patients

TEA remains a gold-standard analgesic technique for major open oncologic thoracic and abdominal surgery, where it provides superior postoperative analgesia, may reduce pulmonary complications, and may facilitate gastrointestinal recovery compared with systemic opioids, although newer regional techniques are increasingly explored as alternatives [14,16,57,68]. As part of an enhanced recovery pathway, TEA has been shown to be safe and to improve postoperative pain control and perioperative comfort in oncologic thoracotomies and laparotomies [78,79,80]. Its greatest clinical value is observed in patients undergoing extensive open procedures associated with substantial postoperative pain and physiological stress [16,57].

Indications for TEA include open esophageal and upper gastrointestinal oncologic surgery, thoracic malignancies, lung cancer, mediastinal tumors, major hepatobiliary and pancreatic cancer surgery, major colorectal cancer surgery performed through an open approach or in high-risk patients, and major open urologic oncologic procedures. TEA may also be particularly beneficial in patients with limited pulmonary reserve or a high risk of postoperative respiratory complications, including elderly patients, smokers, and individuals with chronic obstructive pulmonary disease [COPD]. It may further be considered when opioid-sparing analgesia is desirable, in patients with chronic pain or opioid tolerance, and when a significant surgical stress response is anticipated. This is particularly relevant in prolonged and complex procedures, including cytoreductive surgery with hyperthermic intraperitoneal chemotherapy, surgery for pseudomyxoma peritonei, and retroperitoneal sarcoma surgery [57,80].

Conversely, the routine use of TEA is less strongly supported in minimally invasive surgery, where laparoscopic and robotic techniques are associated with reduced tissue trauma and lower analgesic requirements. In these settings, alternative regional anesthetic techniques and multimodal opioid-sparing analgesia may provide comparable recovery outcomes with fewer hemodynamic adverse effects. Consequently, contemporary practice increasingly favors an individualized approach to TEA based on surgical invasiveness, patient comorbidities, expected postoperative pain burden, and ERAS objectives rather than its routine application in all major cancer procedures [33,37,50,68], Table 2.

## 7. Alternatives to TEA in Major Cancer Surgery

In major cancer surgery, several regional and multimodal analgesic techniques have emerged as alternatives to thoracic epidural analgesia [TEA], particularly when TEA is contraindicated, technically challenging, or associated with clinically significant adverse effects. Thoracic paravertebral block [TPVB] provides unilateral segmental analgesia with pain control comparable to TEA after thoracotomy and breast cancer surgery, while being associated with lower rates of hypotension and urinary retention [38]. Because of its favorable hemodynamic profile, TPVB has become an increasingly attractive alternative for patients in whom extensive sympathetic blockade may be undesirable [36,38].

The erector spinae plane [ESP] block has gained increasing popularity in thoracic, breast, and upper abdominal oncologic procedures because of its technical simplicity, favorable safety profile, and ability to reduce postoperative opioid consumption, although high-quality comparative data against TEA remain limited. Recent studies suggest that ESP block may provide satisfactory analgesia for minimally invasive thoracic procedures while minimizing the risk of hypotension and other neuraxial complications [50].

Quadratus lumborum [QL] and transversus abdominis plane [TAP] blocks are widely used in major abdominal and gynecologic cancer surgery and can provide effective somatic analgesia within Enhanced Recovery After Surgery [ERAS] pathways, although their efficacy for visceral pain is less consistent than that of TEA [33]. Consequently, these techniques are often incorporated as components of multimodal analgesic strategies.

In thoracic surgery, particularly in minimally invasive approaches such as video-assisted thoracoscopic surgery [VATS], intercostal, paravertebral, or ESP blocks combined with multimodal systemic analgesia may achieve comparable postoperative pain control with fewer hemodynamic effects and can be readily incorporated into ERAS protocols [47,50]. Consequently, opioid-sparing multimodal analgesia combining non-opioid agents and regional techniques is increasingly favored to minimize opioid-related adverse effects while maintaining adequate analgesia in patients undergoing complex oncologic surgery, Table 3. The choice of analgesic technique should therefore be individualized according to surgical invasiveness, expected pain severity, patient comorbidities, pulmonary risk, and available expertise [31,74,75] Table 3.

## 8. Clinical Decision Algorithm for Epidural Analgesia

**Figure 1:**
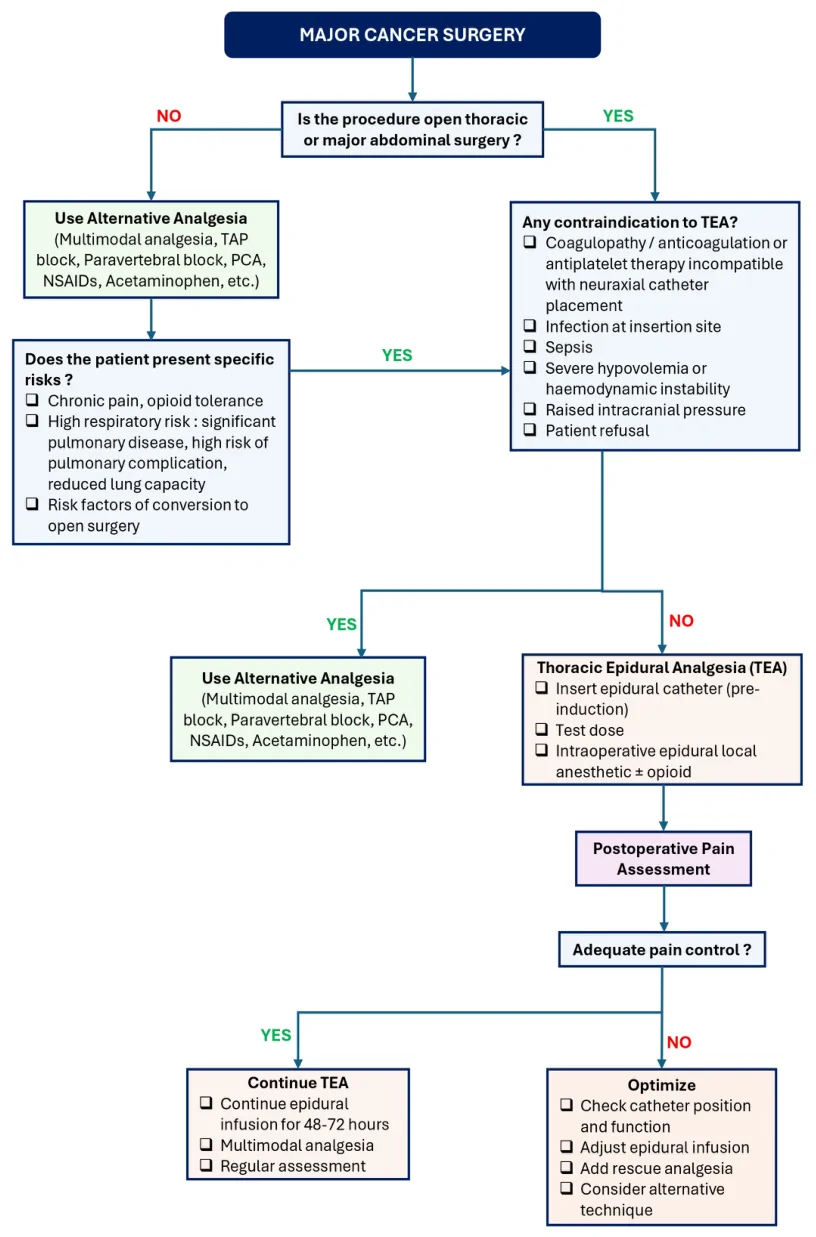
The proposed clinical decision algorithm for selecting thoracic epidural analgesia [TEA] or alternative analgesic strategies in patients undergoing major cancer surgery.

Based on the evidence reviewed in this article, Figure 1 presents a proposed clinical algorithm for postoperative pain management after major open thoracic and abdominal cancer surgery. The algorithm integrates surgical invasiveness, contraindications to neuraxial techniques, patient-specific risk factors, and postoperative pain assessment. TEA is preferentially considered for major open thoracic and upper abdominal procedures in the absence of contraindications, whereas alternative regional and multimodal analgesic techniques may be favored for minimally invasive surgery or when neuraxial blockade is unsuitable. Postoperative reassessment guides continuation of epidural therapy or implementation of corrective and rescue analgesic strategies to optimize recovery within Enhanced Recovery After Surgery [ERAS] pathways [16,57,65,68,74].

## 9. Conclusions

Thoracic epidural analgesia remains one of the most effective techniques for postoperative analgesia following major open thoracic and upper abdominal cancer surgery. Its greatest benefit appears to be in patients undergoing extensive open procedures and in individuals with limited pulmonary reserve, frailty, severe preoperative pain, chronic opioid use, or opioid tolerance. In these settings, TEA provides superior dynamic analgesia, reduces opioid requirements, and may facilitate respiratory recovery, gastrointestinal function, and early mobilization within Enhanced Recovery After Surgery [ERAS] pathways.

However, the role of TEA has evolved considerably with the increasing adoption of minimally invasive surgical techniques and the development of alternative regional analgesic approaches. In laparoscopic, robotic, and minimally invasive thoracic procedures, techniques such as thoracic paravertebral block, erector spinae plane block, and multimodal opioid-sparing analgesia may provide similar postoperative recovery outcomes while reducing the risk of hemodynamic adverse effects [34,37,38,50,68]. Consequently, current evidence supports a selective, patient- and procedure-specific approach to TEA rather than its routine use for all major oncologic procedures. Careful patient selection, meticulous technique, and vigilant perioperative monitoring remain essential to maximize the benefits of TEA while minimizing complications.

Although experimental and observational studies suggest biologically plausible mechanisms through which neuraxial techniques may influence cancer biology, randomized clinical trials have not demonstrated a definitive reduction in cancer recurrence or an improvement in long-term survival attributable to TEA. Its role should therefore remain primarily focused on optimizing perioperative analgesia and functional recovery rather than on modifying oncologic outcomes. Future research should focus on identifying the patients and surgical procedures most likely to derive meaningful benefit from TEA and on defining its optimal integration into current ERAS pathways and multimodal analgesic strategies.

## Figures and Tables

**Table 1:** Evidence Linking Thoracic Epidural Analgesia and Cancer Outcomes. Summary of current evidence regarding local anesthetics, thoracic epidural analgesia [TEA], and cancer outcomes. Experimental studies suggest biologically plausible anticancer effects; however, observational findings remain inconsistent and randomized clinical trials have not demonstrated definitive reduction in cancer recurrence or improvement in survival attributable to TEA [16,33,34,35,36,37,38].

Level of Evidence	Main Findings	Limitations
Preclinical studies	Lidocaine, ropivacaine, and bupivacaine demonstrate anti-proliferative, anti-migratory, and immunomodulatory effects	Many effects occur at supraclinical concentrations or under experimental conditions
Mechanistic clinical studies	TEA may preserve NK cell function, reduce stress hormones, and attenuate inflammatory responses	Surrogate biological endpoints, uncertain translation into survival benefit
Observational studies and meta-analyses	Some studies report reduced recurrence or improved survival	Susceptible to selection bias and confounding
Randomized lung cancer trial	No significant recurrence-free survival benefit	Single disease setting
Randomized colorectal cancer trial	No improvement in disease-free survival	Limited generalizability
Overall interpretation	Biological plausibility exists, but no proven oncologic benefit	Additional high-quality randomized studies are required

**Table 2:** Current role of thoracic epidural analgesia [TEA] according to procedure type and patient risk profile in major cancer surgery. Strong indications are mainly limited to extensive open thoracic and upper abdominal procedures, whereas selective use is recommended for specific high-risk patients undergoing minimally invasive surgery [16,35,57,65,68].

Clinical scenario	Role of TEA	Rationale
Open thoracotomy	Strong indication	Superior dynamic analgesia, improved respiratory mechanics, reduced pulmonary complications
Esophagectomy	Strong indication	Severe postoperative pain, facilitation of pulmonary recovery and ERAS pathways
Major hepatobiliary or pancreatic surgery	Selective indication	Effective analgesia, opioid sparing, enhancement of gastrointestinal recovery
Major upper abdominal laparotomy	Strong indication	Significant postoperative pain burden and stress response
Radical cystectomy and major urologic oncology surgery	Selective indication	May reduce opioid requirements and improve postoperative recovery
Severe COPD or high pulmonary risk	Selective indication	Potential reduction in opioid-induced respiratory impairment
Frailty, sarcopenia, opioid tolerance, chronic pain	Selective indication	Facilitates mobilization and opioid-sparing analgesia
Video-assisted thoracoscopic surgery [VATS]	Alternative techniques often preferred	PVB or ESP block may provide comparable analgesia with fewer hemodynamic effects
Robotic abdominal surgery	Usually not routine	Lower pain burden and effective multimodal analgesic alternatives
Low-risk laparoscopic procedures	Generally, not indicated routinely	Limited incremental benefit compared with multimodal analgesia

**Table 3:** Comparison of Thoracic Epidural Analgesia and Alternative Regional Analgesic Techniques in Major Cancer Surgery. Comparison of thoracic epidural analgesia [TEA] and alternative regional analgesic techniques used in major cancer surgery. The optimal technique depends on the balance between analgesic efficacy, surgical invasiveness, patient comorbidities, pulmonary risk, and recovery objectives. Contemporary practice increasingly favors individualized analgesic strategies rather than routine use of a single technique across all oncologic procedures [34,37,50,68].

Technique	Main Advantages	Principal Limitations	Preferred Indications
TEA	Excellent somatic and visceral analgesia; opioid sparing; improved pulmonary function and gastrointestinal recovery	Hypotension, urinary retention, technical failure, neuraxial complications	Open thoracic surgery, esophagectomy, major upper abdominal surgery
TPVB	Comparable analgesia for thoracic surgery; lower incidence of hypotension and urinary retention	Usually unilateral block; limited visceral analgesia	Thoracotomy, breast surgery, selected VATS procedures
ESP block	Technical simplicity; favorable safety profile; minimal hemodynamic effects	Limited high-quality comparative evidence	VATS, minimally invasive thoracic surgery, selected upper abdominal procedures
QL block	Useful abdominal wall analgesia; ERAS compatible	Less reliable visceral analgesia	Major abdominal and gynecologic surgery
TAP block	Easy to perform; reduced opioid consumption	Primarily somatic analgesia; limited visceral coverage	Lower abdominal surgery and ERAS pathways
Multimodal analgesia	Opioid sparing; flexibility; ERAS integration	Variable efficacy depending on procedure	Minimally invasive surgery and combination strategies

## Data Availability

No new data were created in this study data sharing is not applicable.

## Abbreviations

TEA	Thoracic Epidural Analgesia
PCA	Patient-Controlled Analgesia
TAP block	Transversus Abdominis Plane Block
NSAIDs	Non-Steroidal Anti-Inflammatory Drugs
ERAS	Enhanced Recovery After Surgery
ESP block	Erector Spinae Plane Block
TPVB	Thoracic Paravertebral Block
